# Primary Hydatid Cyst of the Thigh: Atypical Location and Perioperative Strategies to Minimize Recurrence After Accidental Cyst Rupture

**DOI:** 10.7759/cureus.42915

**Published:** 2023-08-03

**Authors:** Hassan A Ahmed, Eid A Almasoudi, Bandar M Hetaimish, Ramy Samargandi

**Affiliations:** 1 College of Medicine, University of Jeddah, Jeddah, SAU; 2 Orthopedic Surgery Department, University of Jeddah, Jeddah, SAU; 3 Orthopedic Surgery Department, Centre Hospitalier Régional Universitaire (CHRU) de Tours, Tours, FRA

**Keywords:** musculoskeletal, cysts, soft tissue mass, soft tissue hydatid, echinococcus granulosus, hydatid cyst

## Abstract

This study presents a rare case of hydatid cyst (HC) located in the left thigh, an atypical site for this parasitic infection, which typically affects the liver and lungs. A 22-year-old female presented with a gradually increasing swelling in the anterior aspect of her left thigh over a period of six months. The diagnosis of the thigh HC was established through a combination of imaging techniques, including ultrasonography and magnetic resonance imaging (MRI), and serological tests. The patient underwent surgical removal of the cyst. We also highlight a management strategy for perioperative accidental rupture of the cyst to minimize the risk of dissemination and reduce the likelihood of recurrence. This report emphasizes the need for a careful multidisciplinary approach to ensure effective diagnosis and successful management of HC, particularly when they occur in atypical locations.

## Introduction

Echinococcus, a cestode belonging to the Taeniidae family, is the source of hydatid diseases, which are cystic parasitic infestations [1]. The organisms can live in both permanent and temporary hosts. The disease is transmitted to intermediate hosts, such as cattle, sheep, humans, goats, horses, and camels, by dogs, wolves, or foxes (the definitive hosts) dispersing eggs via their excretions into the environment [2]. It is endemic in several regions worldwide, including the Mediterranean, Eastern Europe, South America, and the Middle East [3]. The main organs affected by the parasitic cysts are the liver and lung, then the brain, while muscle involvement is a relatively rare occurrence, with an estimated incidence of up to 3% in extra-hepatic cases [3]. Moreover, bones, skeletal and smooth muscles, viscera, and the mediastinum are infrequently affected [4]. Here, we report a case of thigh hydatid cyst (HC) with a comprehensive discussion on its clinical presentation, diagnosis, management, and a review of relevant literature.

## Case presentation

A 22-year-old female patient, with no significant medical or surgical history, presented with left leg pain that caused sleep disturbances. She complained of a gradually increasing swelling in the anterior aspect of her left thigh for about six months. The patient consulted her primary care physician due to the onset of sleep-disturbing pain. There was no history of other systemic symptoms.

**Figure 1:**
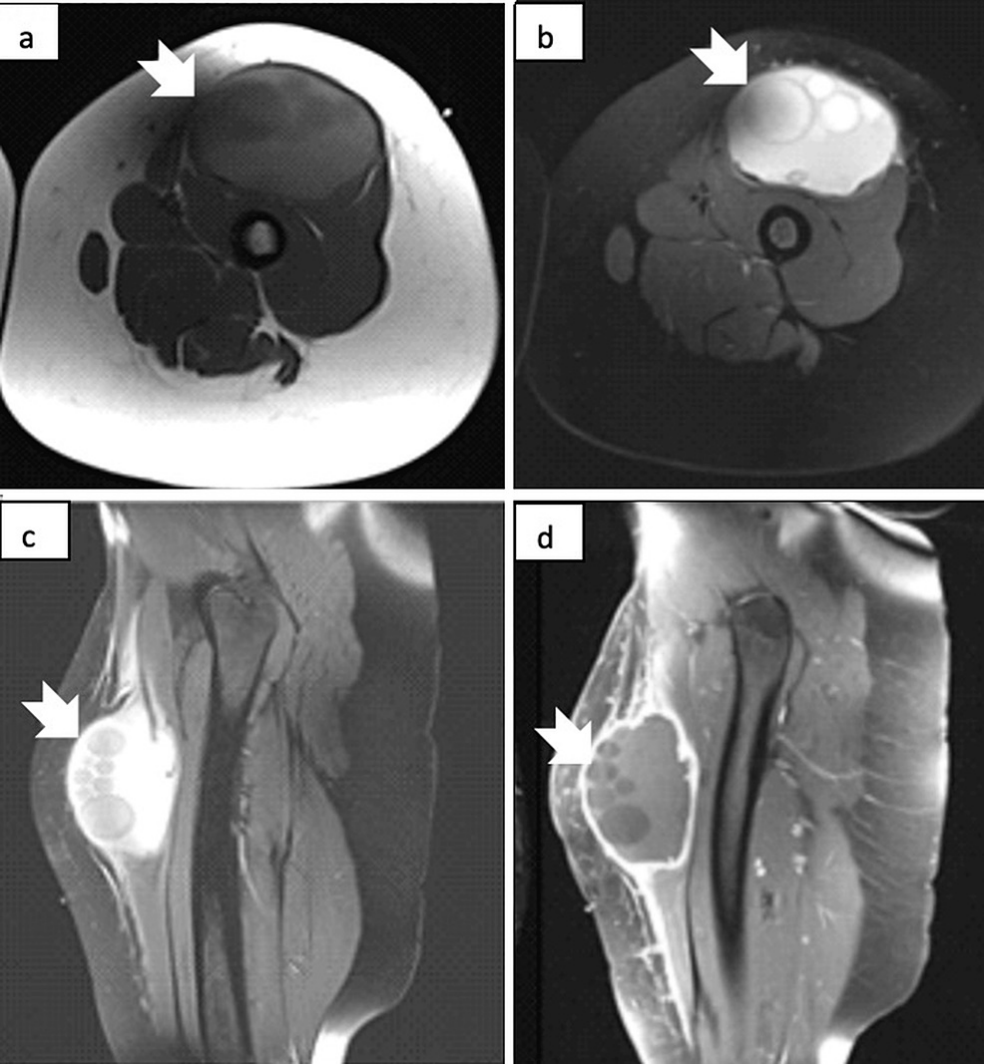
MRI demonstrating the lesion in the left thigh (a) Axial T1-weighted MRI demonstrating multiple daughter cysts as hypointense signals (arrows), relative to surrounding intracystic fluid (white arrow). (b) Axial fat-suppressed T2-weighted image showing cyst formations in the thigh; the daughter cystic wall has a hypointense signal (white arrow). (c) Sagittal fat-suppressed T1-weighted image showing cystic formations in the thigh; the daughter cysts have a hypointense signal (white arrow). (d) T1-weighted image with fat suppression reveals peripheral contrast enhancement after gadolinium injection.

Physical examination revealed a firm, tender, palpable mass at the anterior surface of the left thigh. The lymph nodes were not enlarged, with no other abnormal physical findings. A thigh ultrasound revealed a thick-walled cystic mass deep in the anterior aspect of the proximal part of the left thigh with numerous peripherally arranged internal cysts of various sizes, characteristics suggestive of hydatid disease or a benign neoplastic process. Magnetic resonance imaging (MRI) revealed a thick-walled cystic lesion measuring approximately 8x10x17 cm in the anterior compartment of the thigh (Figure 1). It had internal, peripherally organized tiny cysts and an undulating membrane. There was no sign of underlying muscle infiltration. Hydatid disease was increasingly suspected.

Lung and liver hydatid disease were ruled out by a thoracoabdominal CT scan. The diagnosis was confirmed through serology using the enzyme-linked immunosorbent assay (ELISA) test, which showed a positive result for echinococcus. After confirming the diagnosis, an extensive interrogation was carried out to search for a predisposing cause that led to the development of the lesion. It revealed a history of exposure to dogs during the patient’s childhood, which was around 10 years before the clinical presentation.

**Figure 2:**
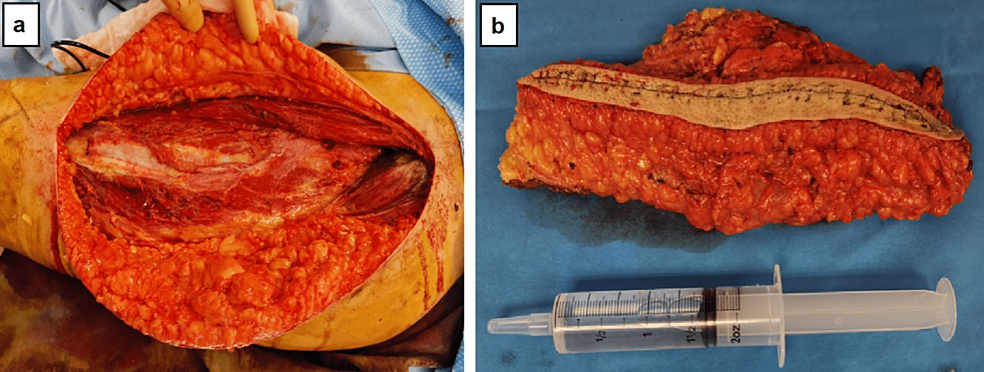
Perioperative photograph (a) Intraoperative view following the removal of the hydatid cyst. (b) Macroscopic appearance of the complete hydatid cyst after surgical excision, including the first incision tract.

Before surgery, albendazole 400 mg twice daily was given orally for four weeks. Then, the patient underwent surgery for total cyst removal. Under general anesthesia, with the patient in a supine position, a longitudinal incision was made in the anterior aspect of the left thigh. An accidental rupture of the cyst occurred during the excision. Immediate closure of the cyst wall was performed to prevent further spillage of cyst contents. Subsequently, the surgical site was lavaged with hypertonic saline to reduce the risk of dissemination. In order to minimize the risk of recurrence following the cyst rupture, a decision was made to close the wound and perform a wide resection of the initial incision tract along with the lesion in a monobloc manner (Figure 2). Preservation of the quadriceps bundle was prioritized; the surgical site was again copiously washed with hypertonic saline and hydrogen peroxide before wound closure. Multiple layers of closure were performed, followed by the application of compression dressing to reduce swelling, promote wound healing, control bleeding, and provide support to the surgical site. The dressing was changed every two days for one week. Macroscopically complete excision with clear negative margins was achieved.

Histopathological reports confirmed thigh hydatid disease with clear margins. Albendazole treatment was continued for three months post surgery with good adherence. Medical follow-ups and consultations were carried on as needed. Post-operative recovery was uneventful, and the patient had no signs of recurrence at the one-year follow-up by CT imaging. The choice of CT imaging for follow-up was made due to the patient's claustrophobia.

## Discussion

It has been reported that subcutaneous HCs account for only 2% or fewer cases among all hydatid diseases in the population [5]. HCs under the skin can be categorized as primary or secondary. In the first category, the presence of at least one HC in typical sites such as the liver, lung, or spleen should be documented [5]. The uncommon incidence of skeletal muscle HC can be explained by the larvae's particular, unfavorable environment caused by the presence of lactic acid and muscular contraction [4]. Myxoma, lipoma, abscess, hematoma, and soft tissue sarcoma are among the differential diagnoses for cystic lesions on the thigh [6]. In this study, primary HC was found in the quadriceps compartment of the left thigh. Many reports in the literature list cases of hydatid disease in the adductors and quadriceps compartments of the thigh [4,5,7-14]. Most patients in such cases were females [4,5,7-13]. The worm can remain dormant for an extended period of time (months to decades) without presenting any particular symptoms of hydatid disease [2]. In this case, exposure to dogs occurred many years before the manifestations appeared. The condition may be discovered incidentally or may cause pressure symptoms that prompt the person to seek medical attention [2]. The HC in this case resulted in tender swelling-induced pain that led to sleep disturbances.

Deep hydatid disease should be diagnosed before surgery, and the sensitivity of serological tests varies depending on the type of hydatid disease [13]. Approximately 90% of individuals with hepatic hydatidosis test positive, while the majority of hydatid diseases in other body parts test negative [13]. Hydatid serology is typically exclusively positive in cyst infections or fissurations [3]. Ultrasound is the most common examination used to diagnose soft tissue swellings, providing information about the fluid character and location of the tumefaction [10]. Hydatidosis can typically be diagnosed by demonstrating a more or less heterogeneous liquid production with the visualization of daughter vesicles, as in our case [6,10]. The preferred diagnostic method in atypical hydatid diseases such as muscular or subcutaneous hydatidosis is MRI, which provides extensive information about the structure and relationships of soft tissues. In the present case, an ultrasound helped with the initial diagnosis which revealed characteristics that might indicate hydatidosis or a benign tumor. The diagnosis of hydatid disease was confirmed by MRI.

HC should be managed based on factors such as size, location, and associated complications. Surgical excision remains the preferred treatment method, and postoperative antihelminthic therapy (albendazole) is administered to prevent recurrence [14,15]. Intraoperative safety precautions involve using fields of hypertonic saline or hydrogen peroxide applied to the wound borders to reduce the risk of recurrence [16-19]. Local recurrence rates may vary, and in cases with inadequate surgical margins or intraoperative cyst rupture, as seen in this case, they could be as high as 12.4% [20].

The management of ruptured HCs requires meticulous intraoperative handling and preventive measures to minimize the risk of dissemination and recurrence. In this case, the focus was on the perioperative management of a ruptured HC. Immediate actions were taken following the accidental rupture of the cyst during excision to minimize contamination. The cyst wall was closed to contain the contents and prevent further spillage. Additionally, the surgical field was washed with hypertonic saline solution to decrease the risk of dissemination. To further mitigate the risk of recurrence after cyst rupture, a decision was made to perform a wide resection of the initial incision tract along with the lesion in a monobloc fashion. This surgical approach aimed to remove any potential residual cyst material and reduce the chances of recurrence. Furthermore, the surgical site underwent thorough washing with hypertonic saline and hydrogen peroxide to ensure effective scolicidal action and eliminate any remaining cystic elements before wound closure. By employing these perioperative measures, the surgical team aimed to effectively manage the ruptured HC, minimize the risk of dissemination, and reduce the likelihood of recurrence. As an adjuvant treatment, albendazole was continued for three months postoperatively.

## Conclusions

The necessity of having a high index of suspicion for HC in individuals with cystic lesions, especially in endemic areas, is highlighted by this case. It is difficult to diagnose a primary HC in the thigh without a combination of imaging techniques and serological tests. For the prevention of complications and the progression of the condition, early diagnosis and effective care are crucial. Having the cyst completely surgically removed along with receiving pre- and postoperative medical care results in a favorable prognosis and considerably lowers the likelihood of recurrence.
